# Proceedings of the St. Louis Medical Society

**Published:** 1898-08

**Authors:** 


### Proceedings of the St. Louis Medical Society.

MEETING OF SATURDAY EVENING, FEBRUARY 5, 1898, PRESIDENT DR. J. C. MULHALL, IN THE CHAIR.

Dr. Keating Bauduy read a paper entitled "Observations on the Treatment of Some Cases of Neurasthenia," written by Jerome K. Bauduy. The doctor also read a paper giving "Microscopical Report," by Dr. C. Fisch. Also "Clinical Report," by himself. (See *Review* of February 26, 1898, page 146.)

DISCUSSION.

Dr. Stoffel.—I would like to ask Dr. Bauduy whether he does not think that the dieting of patients and placing them in hygienic surroundings had not as much to do with the results as his medicine?

Dr. Fairbrother.—I would like to ask another question: If this "Pepto-Mangan" is not in all the class of proprietary medicines?

Dr. Keating Bauduy.—I will endeavor to respond to the questions propounded. Dr. Stoffel wants to know if the dietetic and hygienic measures alone being adopted would not have effected a cure in the cases reported. I will state that in many of these cases we have tried other preparations of iron and with rather negative results; and in all these cases we have observed hygienic and dietetic indications without obtaining these remarkable improvements. Now I do not wish to be understood that this remedy is a panacea; I merely give you the data and clinical facts, and the result of the microscopic investigation, and you can take them for what you believe them to be worth.

I will answer Dr. Fairbrother by saying that I presume that this is a proprietary remedy, but I use a great many other proprietary preparation. I use antipyrine, and I suppose the doctor does; I use phenactine, sulfonal, and other such propietary remedies, and I will tell you candidly, gentlemen, that I use whatever I find benefits my patients. Of course I do not propose to use nostrums or remedies of which we know nothing about their composition. But the Gude preparation of iron does not belong to this class; a great many gentlemen here use it; I use it because it is the best remedy I have obtained for the treatment of these cases.

Dr. Johnston.—There is no iron in it, is there, doctor?

Dr. Keating Bauduy.—Yes, sir, there is iron in it, in the form of a peptonate of iron.

Dr. Jerome K. Bauduy.—One salient feature of this paper which has not been brought out as prominently as it might have been, on which I wish to lay particular emphasis, is that whether it be a proprietary remedy or not, matters not provided it cures our patients. It is our business to cure our patients, it matters not by what means. But the point is this, that it is my opinion, based upon observations in these cases, that we have not paid sufficient attention to the *organic* salts of iron; in other words, that the other preparations of iron do not produce the results that these organic preparations achieve. For years I have used the combination of iron and manganese in daily practice. I have used a great many of these preparations and the great point has been to obtain one which is assimilable, that is elegant, and that will not produce anorexia and other gastric disturbances. Now with the organic salts of iron we have had startling results, and I intend to use them as long as they benefit my patients. I do not wish to be understood by the neurologists and others present as saying that this is a proprer remedy for all cases of neurasthenia, but I do maintain that it is a remedy well suited to those neurasthenic and anæmic cases described, especially those accompanied by hemorrhage. I simply want the gentlemen to judge by the results. "Facts speak louder than words." "*Facta non Verba.*"—*Medical Review*, March 12, 1898.

